# Transient viscous response of the human cornea probed with the Surface Force Apparatus

**DOI:** 10.1371/journal.pone.0197779

**Published:** 2018-05-25

**Authors:** Bruno Zappone, Navinkumar J. Patil, Marco Lombardo, Giuseppe Lombardo

**Affiliations:** 1 Consiglio Nazionale delle Ricerche, Istituto di Nanotecnologia (CNR-Nanotec), Rende (CS), Italy; 2 Dipartimento di Fisica, Università della Calabria, Rende (CS), Italy; 3 Vision Engineering Italy srl, Rome, Italy; 4 Consiglio Nazionale delle Ricerche, Istituto per i Processi Chimico-Fisici (CNR-IPCF), Messina, Italy; University of Michigan, UNITED STATES

## Abstract

Knowledge of the biomechanical properties of the human cornea is crucial for understanding the development of corneal diseases and impact of surgical treatments (e.g., corneal laser surgery, corneal cross-linking). Using a Surface Force Apparatus we investigated the transient viscous response of the anterior cornea from donor human eyes compressed between macroscopic crossed cylinders. Corneal biomechanics was analyzed using linear viscoelastic theory and interpreted in the framework of a biphasic model of soft hydrated porous tissues, including a significant contribution from the pressurization and viscous flow of fluid within the corneal tissue. Time-resolved measurements of tissue deformation and careful determination of the relaxation time provided an elastic modulus in the range between 0.17 and 1.43 MPa, and fluid permeability of the order of 10^−13^ m^4^/(N∙s). The permeability decreased as the deformation was increased above a strain level of about 10%, indicating that the interstitial space between fibrils of the corneal stromal matrix was reduced under the effect of strong compression. This effect may play a major role in determining the observed rate-dependent non-linear stress-strain response of the anterior cornea, which underlies the shape and optical properties of the tissue.

## Introduction

The human cornea is a layer of transparent tissue with the optical function of focusing light onto the retina and the mechanical function of preserving the integrity of the eye. The tissue has an anisotropic microstructure [1–6] mainly comprising a network of collagen fibrils that are intertwined in the anterior stroma (200–250 μm anterior depth) while those in the posterior thirds lie in stacked parallel layers. Under normal conditions, the tissue is subjected to diffuse stresses caused by intraocular pressure and weak localized stresses from external sources, such as eyelid pressure. Knowledge of the native biomechanical properties of human cornea as a function of stress levels and loading rates is crucial to assess the impact of diseases (e.g., keratoconus) or surgery (e.g., riboflavin/UV-A cross-linking; LASIK) [7], and optimize the design of corneal prosthetic devices. [8, 9]. While research rapidly progresses towards *in vivo* characterization of corneal biomechanics, thereby directly measuring the material properties that underlie corneal physiological functions [10, 11], *in vitro* tests can be used to study specific properties and test novel methods of analysis under controlled experimental conditions, particularly under tensile stress that is the physiological condition of the human cornea. [6, 9, 12–20] In strip extensiometry, a tensile force is applied to strips of cornea held between two grips, whereas in bulging and inflation tests the cornea is deformed by increasing the hydrostatic fluid pressure within the whole eye or in artificial anterior chambers. The strain is then measured by means of various methods (digital photography, corneal topography, Scheimpflug imaging, Brillouin microscopy, ultrasonic techniques etc.) and the results are analyzed to derive the modulus of elasticity (*E*). [9, 20–24] There are relatively few studies on the response of human cornea to compressive stresses, particularly on the transient viscous behavior. This may have important implications on tissue function, particularly corneal deformation in response to intraocular pressure (IOP) in disease (e.g., possible role of eye rubbing in the etiology and progression of keratoconus) or after increased removal of tissue by excimer laser or femtosecond laser surgery. [7, 20, 25–30] Moreover, understanding the response of the human cornea to compressive stress may be valuable to widen our understanding on the variability in IOP measurements with applanation tonometry. [31–34] Recently, the compressional response has been studied at sub-micrometric scale with Atomic Force Microscopy (AFM) [2–5, 25] enabling the measurement of both local elastic modulus and viscoelastic properties of human corneal sections at different depths in the stroma. [1, 25]

Each of the above methods has advantages and disadvantages, depending on the tissue properties considered, and the model and approximations used to determine these properties from the mechanical measurements. These experiments have shown large differences in *E* values, ranging from kPa to tens of MPa, mainly due to different testing geometries, techniques and protocols. [9, 20, 22–24] Overall, it has been shown that the stress-strain response of the human cornea is non-linear and rate-dependent, with increasing stiffness for increasing loading rate (stress-stiffening response). [13] In addition, the elastic modulus of the stroma decreases by 40–80% with depth from the anterior one third to the posterior two thirds of the stroma, regardless of the applied compressive or tensile stress. Such non-uniform profile of mechanical properties is highly correlated with a depth-dependent variation of the stromal functional microstructure. [1, 3, 5, 35] In particular, it is known that the anterior portion of the stroma is responsible for maintaining the curvature and optical properties of the human cornea. [36] Any structural or functional change of the anterior stroma may influence the focusing properties of the overall tissue and hence vision.

In the present study, we used a Surface Force Apparatus (SFA) for probing the transient viscous response of the human anterior corneal stroma within physiological pressure levels. The SFA utilizes multiple-beam Fabry-Perot optical interferometry to determine nanoscale variations of the distance between two macroscopic curved solid surfaces with accuracy of the order of the nanometer; it is ideally suited to investigate the mechanical properties of confined transparent biological samples such as the cornea and offers the unique advantage of a non-destructive direct measurement of the sample thickness. Samples can be probed over relatively large areas of several hundred μm^2^ and over time scales sufficiently long to determine the equilibrium behavior of viscoelastic tissues, with pressures between 1 kPa and 1 MPa.

By combining SFA measurements with a pre-existing theory of linear biphasic materials, originally developed to describe the biomechanical behavior of articular cartilage, [37–40] we have determined two key material parameters of the human cornea, namely the elastic modulus and fluid permeability. To the best of our knowledge, based on a thorough literature search, this is the first time that SFA is used for investigating the biomechanics of the human cornea.

## Materials and methods

### 2.1 Corneal tissues

Four corneo-scleral tissues from different human donors, which did not meet requirements for transplantation, were retrieved from the Veneto Eye Bank Foundation (Venezia Zelarino, Italy). Written informed consent from the next of kin was obtained for the use of samples in research. All human tissues were used in compliance with the guidelines of the Declaration of Helsinki for research involving the use of human tissues. The experimental protocol was approved by the Italian National Research Council (CNR) research ethics and bioethics advisory committee. Donors did not have history of corneal pathologies, eye surgery or any major systemic diseases.

All the tissues were cultivated at 30° C in corneal storage medium (MEM HEPES modified solution enriched with nutrients and antibiotics/antimicotics in purified water) for 15–20 days prior to processing. The average donor age was 60±10 years, the average post-mortem interval (from death to culture medium) was 9±7 hours, and the mean endothelial cell density was 2300±70 cells/mm^2^. Each corneo-scleral tissue was de-epithelialized and then dissected using a microkeratome with a 150 μm head (M2 single use, Moria SA, Antony, France) to obtain lenticules of the anterior stroma with targeted thickness, *D*_0_, from 150 μm to 300 μm. Each sample was shipped to the laboratory in 20% dextran enriched storage medium and used within 48 hours from shipment.

### 2.2 Surface Force Apparatus (SFA)

**Fig 1:**
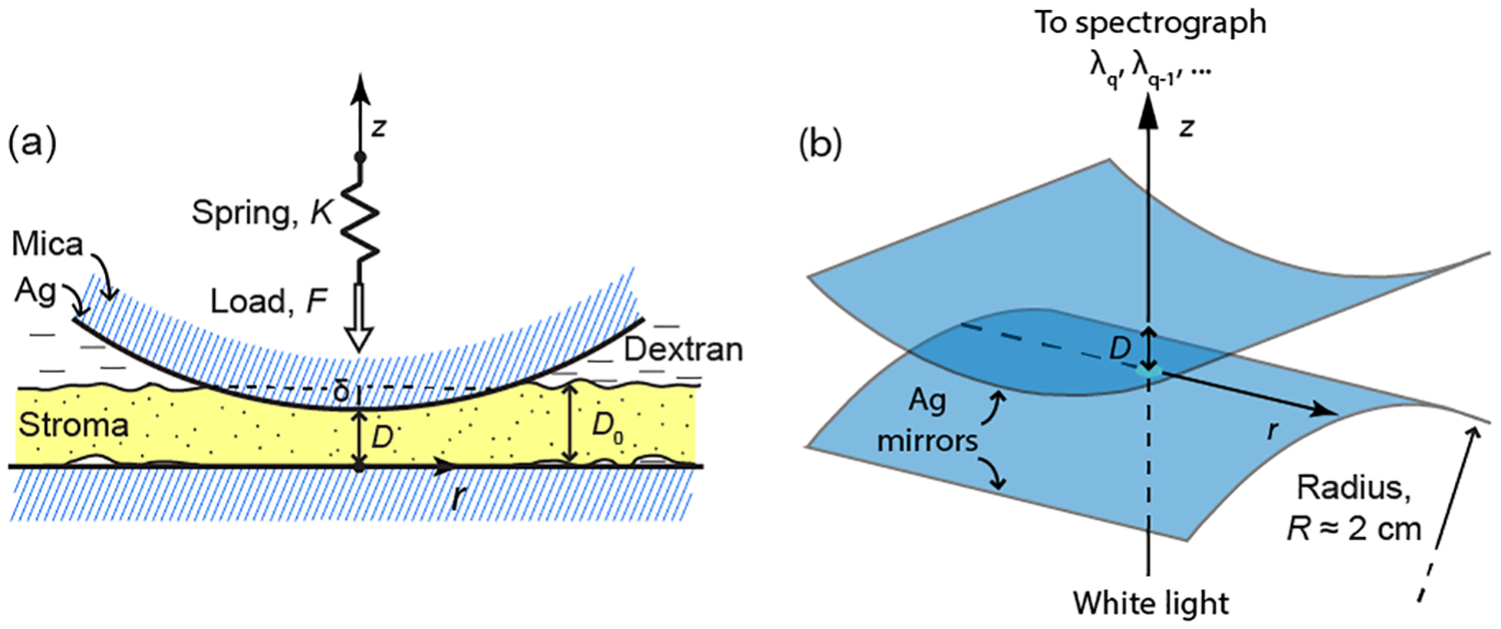
Schematic of the SFA setup for corneal compression and force measurements. (A) Anterior corneal stroma with undeformed thickness *D*_0_ confined between the crossed cylindrical surfaces of the SFA. *r* is the lateral distance from the contact point, where the surface separation distance is *D* and thickness deformation is δ. The normal load *F* is applied via a double-cantilever spring with constant *K* whose fixed end undergoes step-wise vertical displacements (along *z*) as a function of time. (B) Curved Fabry-Perot optical interferometer used to determine *D*.

We used a SFA Mark III (Surforce LLC, Santa Barbara, CA, USA) to measure the normal force (load) *F* generated by a corneal sample with thickness *D*_0_ confined between two crossed cylindrical surfaces with a radius of curvature *R ≈* 2 cm as function of the distance *D* between the cylinders (Fig 1). A detailed description of the technique can be found in previous work. [41] In this study, the corneal sample was placed on the lower cylinder surfaces with its anterior plane facing the opposite cylinder. The sample came into contact with the upper cylinder at a single contact point for *D = D*_0_ eventually widening into a circular contact region as the load *F* was increased and the section compressed to a thickness *D < D*_0_. Since *D*_0_ << *R*, the radius of the contact area was *a* ≈ (π*R*δ)^½^ < (π*RD*_0_)^½^ << *R*, where δ = *D*_0_ − *D* is the deformation (indentation). [42] In the contact area, the corneal thickness *h* was non-uniform and could be approximated as the separation distance between a sphere of radius *R* at a distance *D* from a plane, namely:

$$h\approx D+r^{2}/2R$$ (1)

where *r* < *a* << *R* is the lateral distance from the contact point.

Air bubbles were carefully removed from beneath the corneal sample and the underlying surface. The circular rim of the sample was partially dried with a cotton swab and glued on the surface with a few droplets of cyanoacrylate glue to ensure stability during the force measurements. The corneal stroma was always covered and hydrated with 6% dextran solution.

The force *F* was applied via a double cantilever spring with elastic constant *K* in the range of 250–950 N/m (Fig 1A). The free end of the cantilever was attached to one of the surfaces, while the fixed end was displaced step-wise along the vertical direction, *z*, using a manual micrometer. At the beginning of force measurements, the surfaces were far apart and the force was zero. The surfaces were then approached, eventually compressing (loading) the corneal section, then retracted until the compressive load was completely released. By measuring the change of surface separation distance Δ*D = D*–*D*_0_ as a function of the known cantilever displacement Δ*z = z − z*_0_, the normal force (i.e. along *z*) was determined as *F = K*(Δ*D–*Δ*z*), where it is assumed that *F =* 0 at a sufficiently large distance *D*_0_ and cantilever position *z*_0_. This equation expresses the fact that the cantilever is deflected by an amount Δ*z–*Δ*D* as the surfaces interact with each other during sample compression, thereby producing an elastic force *F*_*s*_ that balances the force generated by surface interactions: *F*_s_ *= —F*. Notice that at large distances Δ*z =* Δ*D*, and therefore the cantilever displacement could be calibrated using the measured distance variation Δ*D*.

The surfaces were approached and retracted using a sequence of displacement steps with fixed length *s* = 10 μm. Each step was applied in less than 4 s and lasted between 1 min and 10 min, corresponding to an average cantilever displacement rate of 0.1–1.0 μm/min. Longer waiting times were used when surface movement was slower. A full cycle of surface approach and retraction took between 40 minutes and 1.5 hour. The maximum strain δ_max_*/D*_0_ reached during compression was 15–20%.

### 2.3 Multiple-beam interferometry with Fringes of Equal Chromatic Order (FECO)

**Fig 2:**
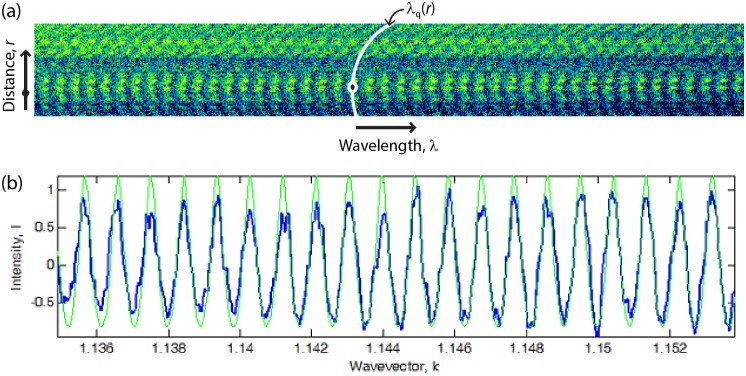
Multiple-beam optical interferometry. (A) Spectrograph showing the light transmitted through a cornea sample as function of the wavelength λ and distance *r* from the point of closest approach (black dot) between the surfaces. Each curved fringe is a FECO with different chromatic order *q* (e.g., white curve λ_q_(*r*)). The curvature along the direction *r* reflects the surface curvature. (B) Comparison between the measured transmitted spectrum at the contact position (blue) and the spectrum calculated from Eq 2 (green).

High-sensitivity multiple-beam Fabry-Perot interferometry was used to determine the surface separation distance *D* (Fig 1). Hard yet flexible sheets of transparent mica, coated on one side with a semi-reflective 540 nm layer of silver, were glued with the uncoated side down on the glass cylinders using a thermosetting glue (EPON 1007 by Shell). The silver coatings acted as the mirrors of a curved Fabry-Perot interferometer and were illuminated with white light collimated along the *z* direction (Fig 1B). The transmittance of a Fabry-Perot interferometer is: [43]

$$T=\frac{1}{1+F{\text{sin}}^{2}(nhk+\alpha )}$$ (2)

where *k* is the wavevector, *F* is the finesse, *n* is the refractive index between the mirrors (*n* ≈ 1.33 for aqueous solutions and highly hydrated corneal tissue) and α is the phase shift upon reflection at the cornea-silver interface. α can be neglected as it is much smaller than *nhk* and slowly varying with *k*. The transmittance is a periodic function of *k* with maxima (peaks) located at:

$$k_{q}=2\pi /\lambda_{q}=(\pi /nh)q$$ (3)

where *q* is the chromatic order and λ the wavelength. *T* was recorded over the spectral range λ = 546–578 nm using an imaging spectrometer (SpectraPro 2300i by Princeton-Acton, Trenton, NJ) coupled to a high-sensitivity high-resolution CCD camera (Newton DU940P-FI by Andor, Belfast, UK). A typical spectrogram showed a set of curved fringes λ_*q*_(*r*), reflecting the curved confinement geometry, each corresponding to a defined chromatic order *q* (Fig 2A). The fringes appeared rough in shape and non-uniform in intensity due non-homogeneous light transmission and scattering through the corneal sample.

The spectrum of light transmitted through the contact point (*r* = 0) showed periodic peaks at wavevectors *k*_q_ given by Eq (3) (Fig 2B) and a Fourier analysis was used determine the distance *D* from the periodicity. Since the spectrum was recorded by the CCD camera at equally spaced wavelength values, the transmittance was sampled over non-linearly spaced wavevectors *k* = 2π/λ. Therefore, the Fourier analysis was done using a Non-Uniform Fast Fourier Transform (NUFFT) algorithm. [44]

**Fig 3:**
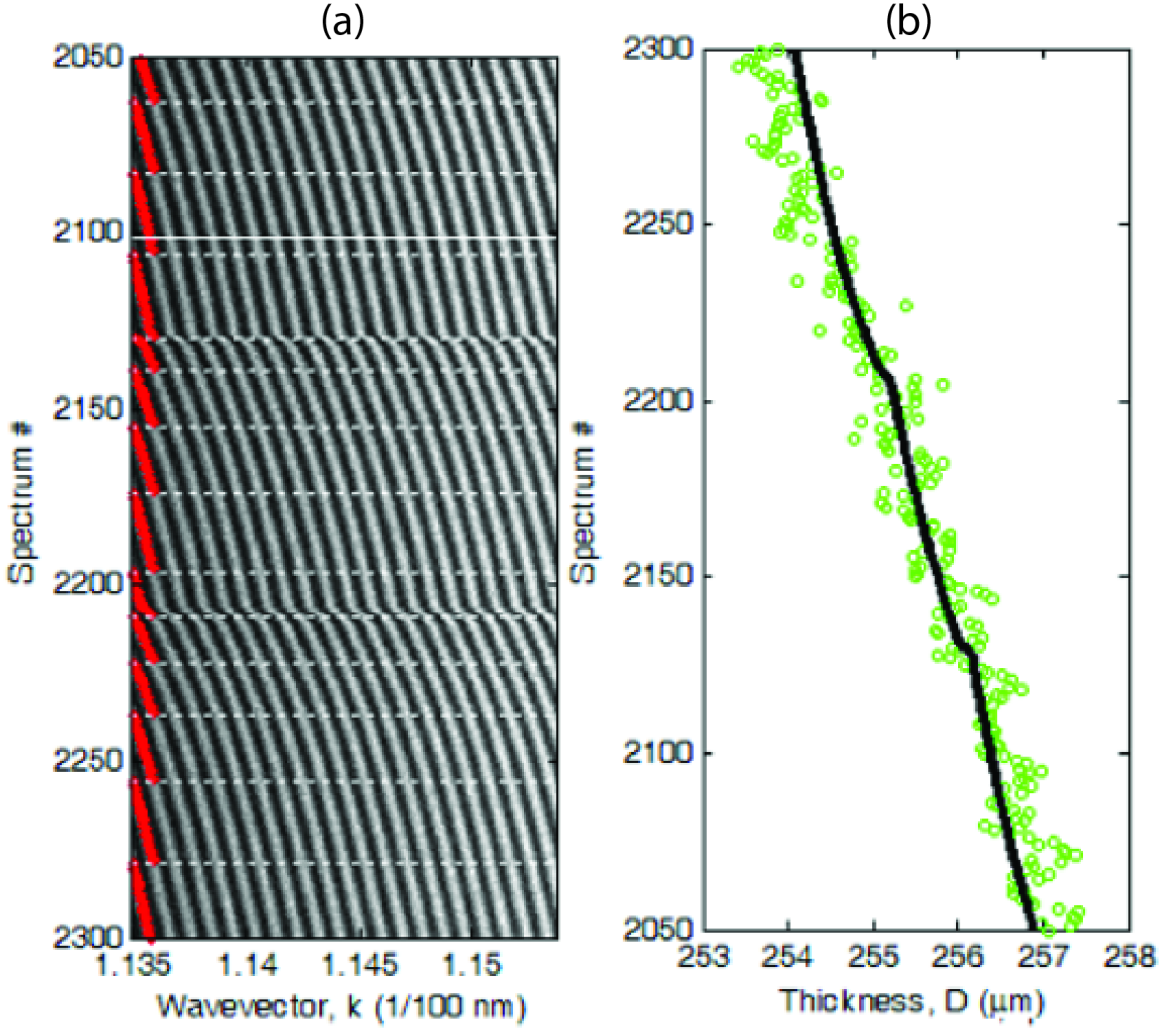
Time-resolved optical measurement of corneal sample thickness. (A) Spectrogram of light transmitted through a corneal sample at the contact point during step-wise surface approach (compression). Each line of the image shows the intensity spectrum recorded at a given time, with a constant rate of 2 spectra/s. Dashed horizontal lines mark the times when a new peak with wavevector *k*_1_ (red dots) entered the spectral range, increasing the chromatic order of the first peak from *q*_1_ to *q*_1_+1. The white solid line corresponds to the FECOs and spectrum of Fig 2. (B) Surface separation distance *D* determined for each spectrum by Fourier analysis (green circles) or by a lock-in computational procedure that tracks the position and order of the first peak (black line).

Fourier analysis has a poor distance sensitivity δ*D* = π/*n*Δ_*k*_ ≈ 5 μm (i.e., it cannot resolve distance variations smaller than δ*D*), where Δ_*k*_ is the wavevector spectral range. On the other hand, multiple-beam interferometry is able to reveal much smaller distance variations, appearing as shifts in the peak positions *k*_q_ (as opposed to peak periodicity, Fig 3B). Therefore, after initially determining *D* via Fourier analysis, we implemented a lock-in computational procedure to track the position *k*_1_ and chromatic order *q*_1_ of the peak with lowest *k* during a cycle of surface approach/retraction. As *D* changed, the peak could either move towards higher *k* and become the second peak, or move towards lower *k* and exit the spectral range, depending whether *D* was decreased (during surface approach) or increased (during retraction), respectively. Fig 3A shows a typical spectrogram, i.e., an image obtained by stacking the spectra acquired at different times interval during surface approach. By monitoring the position *k*_1_ and order *q*_1_ of the first peak, the distance *D* could be measured with high precision (Fig 3B), revealing fine details of the transient viscous tissue response to variations of the applied force.

### 2.4 Analysis of the transient viscous response

**Fig 4:**
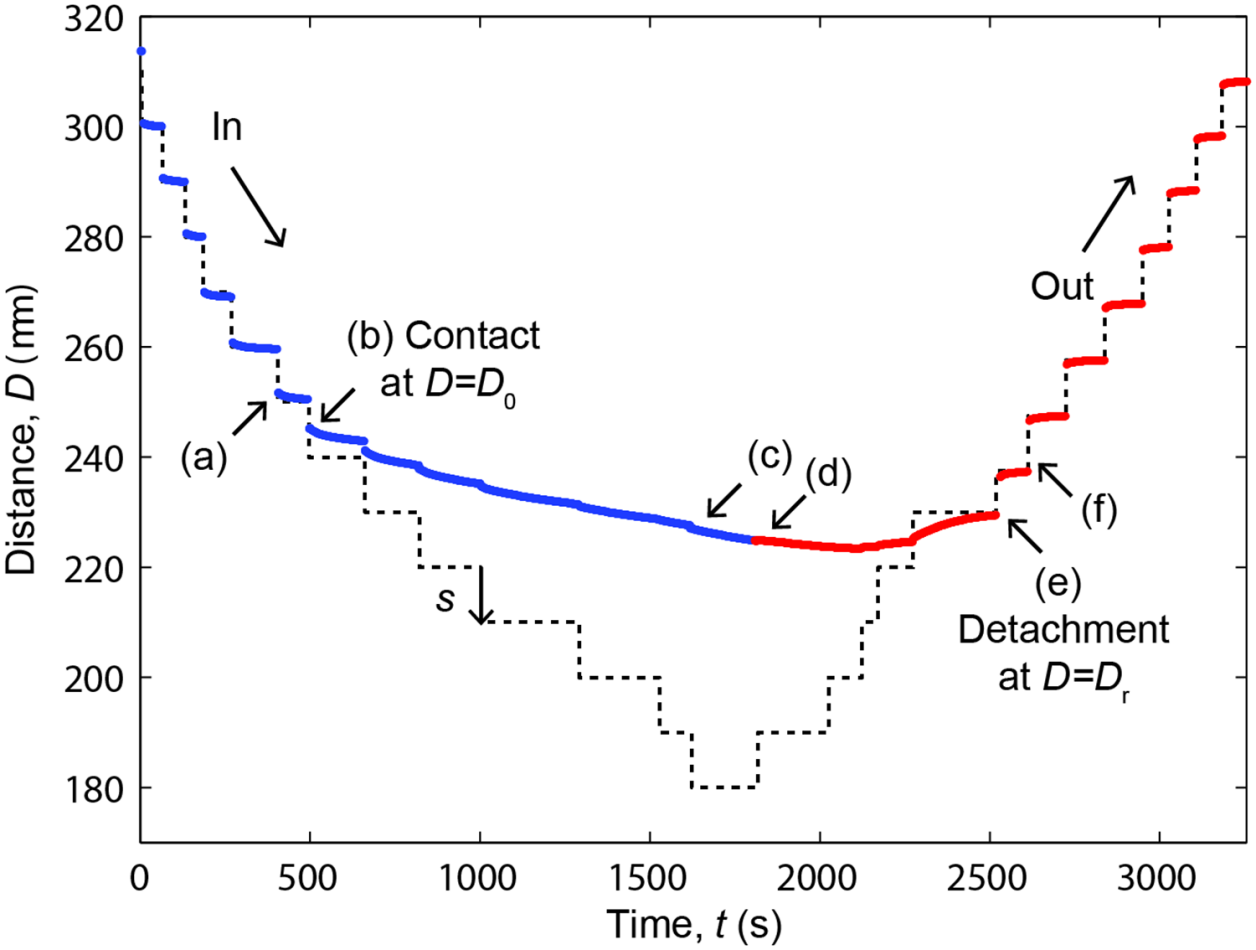
Transient viscous response to a step-wise compression-decompression cycle. *D* is the distance between the silver surfaces (continuous line) in response to step displacements of the cantilever spring with length *s* = 10 μm (dashed line). The surfaces were first approached (‘in’–blue line) then retracted (‘out’–red line). The thickness of the corneal stroma was *D*_0_ before compression and *D*_*r*_ after completely removing the compressive force. Arrows show: (A) last step before surface contact; (B, C) increasing compression with both surfaces in contact with the sample; (D) first step of surface retraction/decompression; (E) **s**urface detachment upon retraction; (F) first step after detachment.

Fig 4 shows the variation of the separation distance *D* between the silver-coated SFA surfaces in response to a series of step displacements with constant length *s* = 10 μm applied to the fixed end of the cantilever. After applying the *k*^th^ step at time *t*_*k*_, the relative surface displacement is *s*_*k*_(*t*) = *D*(*t*)*–D*_*k*_, where *D*_*k*_ = *D*(*t*_*k*_).

We fitted measured values of *s*_*k*_ using exponential functions of the type *f*_*k*_ = *A*_*k*_{1 − λ_*k*_ exp[−(*t* − *t*_*k*_) / τ_*k*_} that describe the viscoelastic creep response of a standard linear solid (with single element). Namely, the relative displacement after applying *k*^th^ steps was fitted with the cumulative exponential function:

$$s_{k}=\sum_{i=1}^{k}f_{i}(t)$$ (4)

where each *f*_*i*_ is a single exponential of the type shown above. Eq (4) takes into account that the displacement *s*_*k*_ relative to the *k*^th^ step may not reach the equilibrium amplitude before the *k*+1^th^ step is applied. Therefore the effect of steps of order *i<k* can persist and cumulate into the response to the *k*^th^ step. The exponential function *f*_*k*_ in Eq 4 represents the response to the very last step of a series of *k* consecutive steps and was determined by fitting the quantity $s_{k}-\sum_{i=1}^{k-1}f_{i}(t)$.

Each response function *f*_*k*_ in Eq (4) includes an immediate elastic response with amplitude *A*_*k*_(1−*λ*_*k*_) at time *t = t*_*k*_, a transient exponential response with a relaxation time *τ*_*k*_, and an equilibrium response with amplitude *A*_*k*_ for *t >> t*_*k*_ + *τ*_*k*_. The value (1−*λ*_*k*_), corresponding the ratio between the immediate and equilibrium amplitudes, characterizes the viscoelastic behavior of the material considered. For *λ*_*k*_ = 0 the material is purely elastic and instantaneously reaches the equilibrium deformation *A*_*k*_ without showing any transient viscous response. For *λ*_*k*_ = 1 the material is purely viscous and shows a long-term viscous response without instantaneous deformation. For values 0 < *λ*_*k*_ < 1 the material response is viscoelastic.

The total deformation measured after application of *k* steps is $\delta_{k}(t)=D_{0}-\sum_{i=1}^{k}s_{i}(t)$ and the total strain is δ_*k*_(*t*) = ε_*k*_(*t*)/*D*_0_. After fitting *s*_*k*_ with Eq 4, the total equilibrium deformation can be calculated as $\delta_{e,k}(t)=D_{0}-\sum_{i=1}^{k}A_{i}$. Also note that δ_*k*_ < 0 for *D > D*_0_ (i.e., before surface contact).

## Results

**Fig 5:**
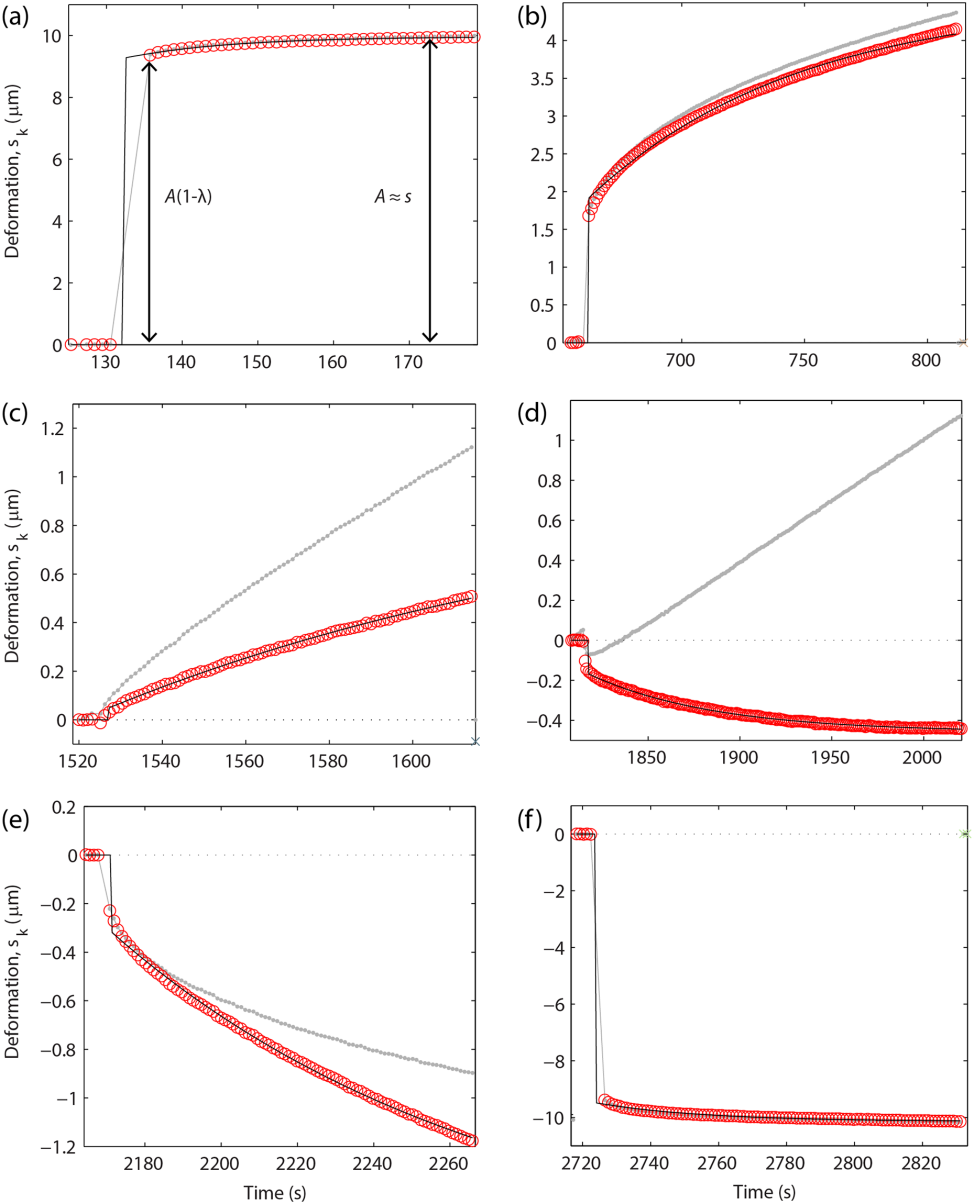
Cumulative exponential fit of the transient viscous response. Gray dots show the relative surface displacement *s*_*k*_ = *D − D*_*k*_ measured after applying *k* displacement steps with amplitude *s* = 10 μm to the SFA cantilever. When both surfaces were in contact with the sample, *D* and *s*_*k*_ were respectively the sample thickness and relative deformation after applying *k* steps. The relative displacement caused only by the last *k*^th^ step (red circles) was obtained after subtracting the cumulative response of the previous *k* − 1 steps (Eq 4) and was fitted with a single exponential (solid line). (A) Surface approach before contact. (B, C) Increasing compression. (D) First step of surface retraction/decompression. (E) Surface detachment. (F) Retraction after detachment.

When the corneal sample was not deformed (i.e. not in contact with both surfaces), the relative surface displacement *s*_*k*_ reached the equilibrium amplitude *s* after a very short response time *τ*_*k*_ < 10 s with a negligible viscosity factor *λ*_*k*_ < 0.1. This behavior was observed both before surface contact (Fig 5A) and after completely separating the surfaces (Fig 5F), showing that the surfaces moved almost freely in dextran solution in response to cantilever displacements. The presence of a short response time was due to small mechanical drifts of the micrometer as well as viscous forces generated by the dextran solution as it flowed out of the contact during surface approach or into the contact region during separation.

When the cornea was compressed for the first time upon approaching the surfaces to a distance *D ≤ D*_0_, the transient response became slower and the equilibrium amplitude *A*_*k*_ was smaller than *s*, revealing the presence of significant repulsive forces (Fig 5B). The response time was much longer than the interval between consecutive cantilever step displacements and we used the cumulative exponential fit (Eq 4) to determine the equilibrium amplitude *A*_*k*_, viscous factor *λ*_*k*_ and relaxation time *τ*_*k*_. In fact, the sample thickness *D* continued decreasing even after reversing the sense of cantilever displacement (Fig 5D). The validity of Eq 4 is demonstrated by noting that the displacement $s_{k}-\sum_{i=1}^{k-1}f_{i}(t)$, caused only by the very last step of a series of *k* steps, immediately changed sign upon reversing the cantilever displacement (Fig 5D). Upon further retraction the surfaces lost mechanical contact, i.e., the equilibrium force decreased to zero, at a distance *D*_*r*_ *< D*_0_ indicating that the anterior cornea did not recover its original thickness and remained partially compacted after compression.

**Fig 6:**
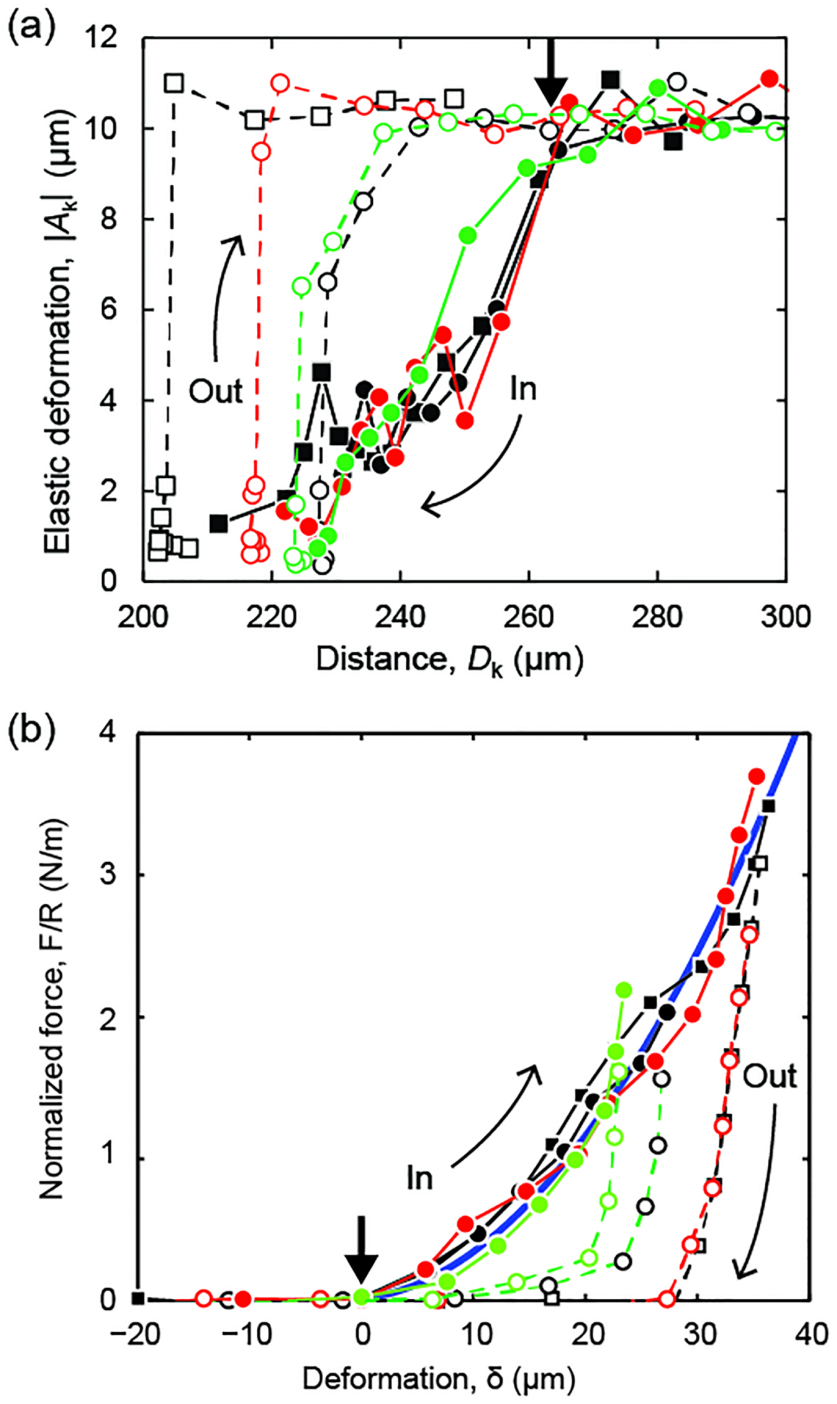
Equilibrium response. (A) Amplitude *A*_*k*_ of the elastic deformation in response to the last step of a series of *k* cantilever displacements. *D*_*k*_ was the surface separation distance when the last step was applied. (B) Elastic force *F* normalized by the curvature radius *R* as a function of the total equilibrium deformation δ. The data and symbols are the same as above. The thick solid line is a model curve *F/R* = π*E*δ^2^/*D*_0_ with *E* = 0.2 MPa. Filled and open symbols indicate surface approach (in) and retraction (out), respectively. Squares/circles indicate different contact positions. Different colors indicate different measurements. The bold arrow indicates the undeformed thickness *D*_0_ = (265 ± 10) μm.

Fig 6A shows the equilibrium amplitudes *A*_*k*_ of the fit exponentials (Eq 4) as a function of the surface distance *D*_*k*_ for a series of consecutive measurements carried out on a representative corneal sample. When the cornea first came into contact with the upper surface, the value |*A*_*k*_| of surface displacement decreased below *s* due to the presence of a repulsive force. This occurred for *D*_*k*_ < *D*_0_ and therefore allowed to determine the undeformed section thickness (*D*_0_ = (265 ± 5) μm in the case of Fig 5). The equilibrium amplitude decreased by one order of magnitude as the cantilever spring was progressively loaded and the distance reached the maximum strain *ε* = 1−*D*/*D*_0_ ≈ 15% attainable with the SFA (*D ≈* 220 μm in the case of Fig 5).

When the surfaces were retracted, the amplitude remained small until the surface contact was lost (i.e., the amplitude was again *A*_*k*_ *= s*) at a surface distance *D*_*r*_ *< D*_0_. The incomplete thickness recovery was due to the fact that the cantilever displacement rate (0.1–1.0 μm/min) was faster than the rate of recovery of the corneal section. This behavior is evident also in Fig 6B showing the equilibrium force *F*_e_ determined from the equilibrium deformation δ_*e*,*k*_.

The force was normalized by the surface radius of curvature *R* = 2 cm to allow comparison with measurements obtained with other techniques and geometries. [1, 25, 45] The force was repulsive during both surface approach and retraction. However, it rapidly decreased to zero as the surfaces were separated, consistent with the observation that mechanical contact was lost at a distance *D*_*r*_*<D*_0_. The cornea did recover its undeformed thickness *D*_0_ after leaving the surfaces well separated for more than 40 minutes, as shown by subsequent measurements on the same position (Fig 6A).

The equilibrium repulsive force generated by an elastic section compressed between a rigid sphere and plane can be calculated using a simple “elastic foundation” model [42]:

$$F_{e}/R=\pi E\delta_{e}^{2}/D_{0}$$ (5)

where *E* is the elastic modulus. Eq (5) describes reasonably well the experimental force curves obtained upon approaching the surfaces, with values of *E* varying between 0.17 MPa and 1.43 MPa (0.47±0.46 MPa). Note that SFA measurements also provided measurements of the out-of-equilibrium deformation δ(*t*) and force *F*(*t*) at any time *t* (not shown). Applying Eq (5) to out-of-equilibrium conditions gives higher and rate-dependent values of the (apparent) elastic modulus *E* since, for a given cantilever displacement, δ is smaller and *F* is higher than at equilibrium. The maximum force attained in our experiments was *F* ≈ 0.14 N, which corresponded to an average pressure *P = F/A* ≈ 32 kPa over a circular contact area *A* = 2*πR*δ = 4.4 mm^2^. [42] Higher pressure can be obtained using smaller values of *R* and more rigid cantilever springs. [41]

**Fig 7:**
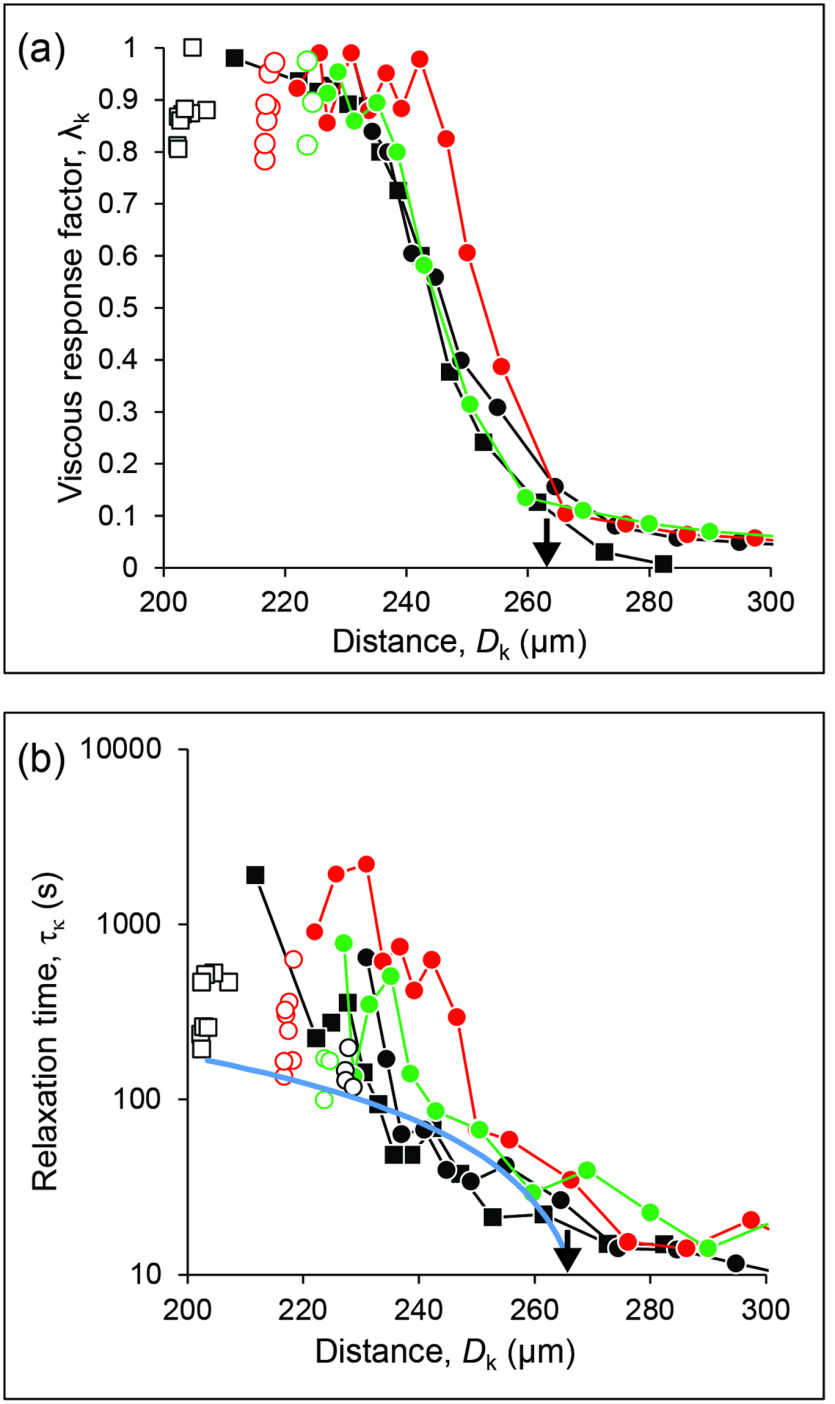
Viscoelastic response. (A) Variation of the viscous response factor *λ*_*k*_ to changes of surface distance *D*_*k*_. (B) Variation of the relaxation time *τ*_*k*_. The solid line is the curve τ = *c*δ with *c* = 2.35×10^6^ s/m. The data and symbols are the same as in Fig 6. Data obtained after surface detachment have been omitted for clarity.

Fig 7A shows that the viscoelastic response of the anterior cornea changed during compression from predominantly elastic (*λ*_*k*_ = 0) to purely viscous (*λ*_*k*_ = 1) as the strain δ/*D*_0_ was increased from zero to less than 10% (corresponding to *D* ≈ 240 μm). This was correlated to an increase of relaxation time *τ*_*k*_ by almost two orders of magnitude, reaching values as high as 15 minutes (Fig 7B).

## Discussion

In this study, we used SFA for assessing the compressional response of the human anterior corneal stroma. The values of the elastic modulus *E* = 0.17–1.43 MPa determined by fitting the compressive force vs. deformation curves (Fig 6B) were within the range of values reported in the literature: 0.01–10 MPa. Such large variability of *E* is primarily due to differences among species (human, porcine, bovine, etc.), test geometry and experimental conditions (i.e., type of mechanical testing, e.g., tensile, compressive or inflation, applied pressures and deformation rates, level of cornea hydration, thickness of the cornea samples, etc.). On the other hand, the anisotropic microstructure of the cornea with collagen fibrils that are intertwined in the half anterior stroma and oriented primarily parallel to the surface in the half posterior stroma is expected to differentiate the elastic moduli measured in or out of the plane of a corneal section. Indeed, values exceeding 1 MPa are commonly found in strip extension and inflation tests on thick samples of human and porcine corneas undergoing (in-plane) stretching deformation, [46] whereas values as small as a few kPa have been reported for (out-of-plane) compression tests on porcine cornea. [4, 7] The likely explanation of the intermediate values of *E* found in our study is that compressing the anterior corneal stroma between the SFA surfaces creates both in-plane and out-of-plane deformations due to the pronounced interweaving of collagen fibrils in the corresponding region. The lowest values of *E* were measured in whole-cornea unconfined compression test where the sample expands laterally with low friction at the confining surfaces as the (normal) compressive load is applied. [7, 27] In our experiments, the cornea was in contact with a silver-coated surface that may not be smooth enough to allow slippage and unconfined lateral expansion. In this case, an additional repulsive force could be generated, directed radially towards the contact position, and effectively increase the resistance to compression. Although the elastic modulus of the cornea determined under compressive stress may somewhat be less informative than tensile data as to the specific tissue properties and function under physiological condition, nevertheless its knowledge may be valuable for improving our understanding on particular pathological conditions, such as keratoconus, and for assessing applicability of the accepted law of applanation tonometry. [1, 28, 30, 32]

The transient viscous relaxation observed in our experiments can be explained in the framework of the linear biphasic theory that was formerly developed for the study of cartilage and other soft tissues, [37–40] and recently applied to characterize the viscoelastic response of porcine cornea [7, 27, 47]. The material is considered as a mixture comprising a deformable porous network of elastic solid material permeated by an incompressible viscous fluid. In the corneal stroma, the solid network mainly comprises collagen fibrils and proteoglycan core proteins, while the fluid fraction is a water solution enriched of glycosaminoglycans. The glycosaminoglycan side chains form inter-fibrillar bridges between collagen fibrils and keratocytes are embedded within this matrix. [47–49] When an external stress is applied to the tissue, the fluid is transiently pressurized in the contact region and flows through the interstitial space between fibrils of the corneal stromal matrix towards undeformed regions with zero (ambient) pressure. The solid network is deformed by the external stress as well as the transient fluid pressure, both of which contribute to the instantaneous (apparent) elastic modulus, whereas the elastic modulus *E* of the solid network determines the equilibrium deformation. The fluid flow is governed by Darcy’s law: *q* = (*k*/η)Δ*P*/*a* where *q* is the flow velocity (in m/s) between regions with pressure difference Δ*P* separated by a distance *a*, *k* is the intrinsic permeability of the porous material and η is the fluid viscosity. A simple dimensional analysis provides a scaling law for the time needed to equilibrate the pressure difference: τ = (η/*k*)*a*^2^/*E*. In the SFA geometry, the separation distance *D* is much smaller than the radius of curvature *R* and, during compression, fluid flow is directed radially outward from the contact area towards unpressurized regions. In this geometry, the radius *a* of the circular pressurized contact region is such that *a*^2^ ≈ *2Rδ*, where *δ* = *D*_0_ − *D* is the deformation. Therefore, the relaxation time due to viscous fluid flow is expected to increase linearly as the tissue is compressed, namely τ ≈ *c*δ where *c =* 2(η/*k*)*R*/*E*. We stress that poro-elasticity naturally captures the dependence of relaxation time τ on contact radius *R* and deformation *δ*. Namely, the distance *a* for the fluid to travel to reach the unpressurized reservoir increases as *R* and δ increase. In contrast, with linear viscoelasticity one would have to recalibrate the time constant of the viscoelastic solid model as a function of *R* and thickness *D*_0_ [50]. Detailed calculations for plane-plane compression geometry [27, 38, 40] and spherical nano-indentation [51] support the validity of the dimensional analysis. The experimental data followed the expected behavior for small strain values δ/*D*_0_ < 10% (Fig 7B, solid line), with a slope *c ≈* 2×10^6^ s/m. Considering the elastic modulus determined from the force curve (Fig 6B with average value *E ≈* 0.45 MPa), a fit to the data gives a permeability η/*k* ≈ 10^−13^ m^4^/(N∙s), which is comparable to the values reported for human [51] and porcine [27, 47] corneas.

For larger strain values, the relaxation time increased more than expected as the thickness was decreased. The likely cause of this behavior is that compression and compaction of the human cornea entail a reduction of the average inter-fibrillar space and compaction of the lamellae in the stromal matrix and therefore a decrease of porosity and permeability. [47] A similar effect has been observed in the compression of human cartilage [37] and porcine cornea [27]. The decrease of permeability at high strains has implications in the stress-strain response and may explain the observed non-linearity and rate dependence of the elastic modulus reported in the literature, with increasing stiffness for high loading rate (stress-stiffening response). [13]

The present SFA study of human corneal transient viscous response has two main limitations. First, the macroscopic crossed-cylinder geometry of the SFA has not been theoretically considered in the context of biphasic theory and analytical expressions are not available for the relations between strain and time, force and strain, or relaxation time and strain. Therefore, the material parameters calculated from our experimental data may be affected by the simplified models and scaling relations adopted for our analysis. Some measured quantities, such as the viscous factor λ, cannot be readily linked to material parameters, although they are expected to be related to the rate dependence of the elastic modulus. Second, the friction between the corneal sample and the surfaces was not measured or controlled. The cornea may partially slip or adhere to the surfaces, depending also on the applied normal load. It is interesting that friction measurements can also be carried out in the SFA using different surface types, as done for cartilage samples [52].

Resolving the viscoelastic response of the corneal tissue as a function of time with the SFA may provide valuable information on the influence of eye rubbing in the development or progression of keratoconus, which is the primary cause of corneal transplantation in young adults. In addition, SFA can be used to assess the efficacy of riboflavin/UV-A corneal cross-linking treatment, which aims at halting the progression of keratoconus by irradiating the cornea with UV-A after stromal soaking with riboflavin. The procedure is expected to increase the mechanical tissue strength via creation of additional cross-linking bonds between stromal proteins. In previous work using AFM, [25] we have demonstrated both an increase of the elastic modulus and a decrease of hysteresis after riboflavin/UV-A cross-linking at the scale of stromal molecular interactions. Adding information about how the induced treatment changes the mechanical properties and transient viscous response of the anterior human cornea (i.e., the part of tissue that is primarily changed by treatment) with SFA may have significant clinical implications. While *in vivo* tests make progress towards a full characterization of corneal biomechanics in physiological conditions, [10, 11, 53, 54] SFA may serve as a tool for the *in vitro* testing of specific corneal biomechanical properties and treatments, particularly to quantify hydraulic permeability and riboflavin/UV-A cross-linking action, at a scale comparable with *in vivo* tests.

## Conclusions

Using the SFA we directly measured with an optical technique the time-dependent deformation of the corneal thickness and characterized the repulsive force produced in response to a step-wise cycle of compression and decompression between two crossed-cylinder macroscopic surfaces. We estimated the elastic modulus *E* and the relaxation time τ; from these quantities, we determined a constitutive material parameter for describing the viscous response of the cornea, namely the hydraulic permeability. Careful analysis of the thickness deformation, taking into account the long relaxation time and cumulative effect of multiple loading/unloading steps, allowed us to determine the main parameters characterizing the response to the *k*^th^ step, namely the instantaneous and equilibrium deformation, and the relaxation time, all of which are thickness-dependent. Using a simple foundation model, we determined the equilibrium force due to elastic deformation of the solid fraction (i.e. anisotropic collagen fibril network) of the corneal tissue, from which the equilibrium compression modulus could be determined. The most important result was that the relaxation time vs. thickness curve could be fitted with a simple scaling law to determine the fluid permeability of the undeformed cornea. The permeability was found to be strain-dependent and rapidly decreasing for strain values exceeding 10%, suggesting a contraction of the inter-fibrillar space of the stromal matrix. This behavior affects the transient viscous stress-strain response and may contribute to the observed non-linear, rate-dependent response, with increasing stiffness at increasing loading rate (stress-stiffening response). [13]
